# Cloning, Expression Analysis, and Molecular Modeling of the Gamma-Aminobutyric Acid Receptor Alpha2 Subunit Gene from the Common Cutworm, *Spodoptera litura*

**DOI:** 10.1673/031.013.4901

**Published:** 2013-06-04

**Authors:** Hongliang Zuo, Lu Gao, Zhen Hu, Haiyuan Liu, Guohua Zhong

**Affiliations:** Laboratory of Insect Toxicology, South China Agricultural University, Guangzhou 510642, China

**Keywords:** developmental expression, GABA receptor, gene structure, three-dimensional model, tissue distribution

## Abstract

Intensive research on the molecule structures of the gamma-nminobutyric acid (GABA) receptor in agricultural pests has great significance to the mechanism investigation, resistance prevention, and molecular design of novel pesticides. The GABA receptor a2 (*SlGABARα2*) subunit gene in *Spodoptera litura* (Fabricius) (Lepidoptera: Noctuidae) was cloned using the technologies of reverse transcription PCR and rapid amplification of cDNA ends. The gemonic DNA sequence of *SlGABARα2* has 5164 bp with 8 exons and 7 introns that were in accordance with the GT-AG splicing formula. The complete mRNA sequence of *SlGABARα2* was 1965 bp, with an open reading frame of 1500 bp encoding a protein of 499 amino acids. The GABA receptor is highly conserved among insects. The conserved regions include several N-glycosylation, Oglycosylation, and phosphorylation sites, as well as 4 transmembrane domains. The identities that *SlGABARα2* shared with the GABA receptor a2 subunit of *Spodoptera exigua, Heliothis virescens, Chilo suppressalis, Plutella xylostella, Bombyx mori* ranged from 99.2% to 87.2% at the amino acid level. The comparative 3-dimensional model of SlGABARα2 showed that its tertiary structure was composed of 4 major α-helixes located at the 4 putative transmembrane domains on one side, with some β-sheets and 1 small α-helix on the other side. SlGABARα2 may be attached to the membrane by 4 α-helixes that bind ions in other conserved domains to transport them through the membrane. The results of quantitative real time PCR demonstrated that *SlGABARα2* was expressed in all developmental stages of *S. litura*. The relative expression level of *SlGABARα2* was the lowest in eggs and increased with larval growth, while it declined slightly in pupae and reached the peak in adults. The expressions of *SlGABARα2* in larvae varied among different tissues; it was extremely high in the brain but was low in the midgut, epicuticle, Malpighian tube, and fat body.

## Introduction

Gamma-aminobutyric acid (GABA) is an inhibitory neurotransmitter in vertebrates and invertebrates that can suppress neurotransmission, hyperpolarize postsynaptic membrane, decrease ion inflow, and slow down cellular metabolism, a process that results in the neuron of postsynaptic membrane being brought into a protective inhibitory condition (Zhao et al. 2005; Ju et al. 2010). The GABA receptor in insects has been intensively explored, and remarkable progress has been made in recent years (Darlison et al. 2005; Casida and Tomizawa 2008). It is an oligomeric protein, its molecule weight is 220–400 kD, and it is composed of 5 different subunits: α, β, γ, δ, and ρ. Every subunit has 4 transmembrane domains (TM1, TM2, TM3, and TM4), and the 5 subunits are linked with the hydrophobic ion channel by the TM2 domains to form a chloride ion channel (Chang and Weiss 2000; Ozoe and Akamatsu 2001; Johnston 2005). In terms of gene structure, several kinds of gene sequence of GABA receptors have been cloned from insects whose complete genome sequence have been identified, such as *Drosophila melanogaster, Bombyx mori, Triboloum castaneum*, and *Anopheles gambiae* (Chen et al. 1994; Miyazaki et al. 1995; Holt et al. 2002; Yu et al. 2010).

The GABA receptor is a major target of a series of important insecticides, such as cyclopentadiene and abamectin. However, resistance to these pesticides is getting more serious in several pest species (Wang and Wu 2007; Chen et al. 2011). The resistance to pesticides was mainly caused by the mutation of target genetic structures in pests (Zeineb et al. 2008; He et al. 2009). It was shown that the mutation of the GABA receptor in *Anopheles funestes, Drosophila simulons*, and *Sogatella furcifera* can significantly enhanced their resistance to the traditional pesticides (Gaelle et al. 2005; Lu et al. 2009; Nakao et al. 2010; Wondji et al. 2011). At present, the structures of the GABA receptor have been isolated from only a few species, for instance, *Spodoptera exigua* (Shang et al. 2009), *Plutella xylostella* (Yuan et al. 2010), and *Laodelphax striatellus* (Narusuye et al 2007). The vast majority of GABA receptors in different agricultural pests remain to be identified. Therefore, intensive research on the molecule structures of the GABA receptor in agricultural insects has great significance for research of pesticides, resistance prevention, and novel pesticide invention.

The common cutworm, *Spodoptera litura* (Fabricius) (Leidoptera: Noctuidae), is a prevalent agricultural pest in China, causing enormous economic losses due to its wide range, rapid reproduction, and resistance to pesticides (Huang et al. 2006). Chemical pesticides are still the main method for controlling *S. litura* all over the world (Ahmad et al. 2008). However, the resistance of *S. litura* to traditional pesticides is getting more and more serious (Shad et al. 2010). Analysis of genetic structure and expression pattern of the GABA receptor in *S. litura* may provide a new strategy to suppress the resistance of pests and increase the efficiency of pesticides. In this study, cDNA of gamma-aminobutyric acid receptor a2 subunit in *S. litura* (named as *SlGABARα2)* of a laboratory susceptible strain was amplified via RT-PCR, and developmentand tissue-specific expression patterns were analyzed using quantitative real-time PCR (qRT-PCR).

**Table 1.**
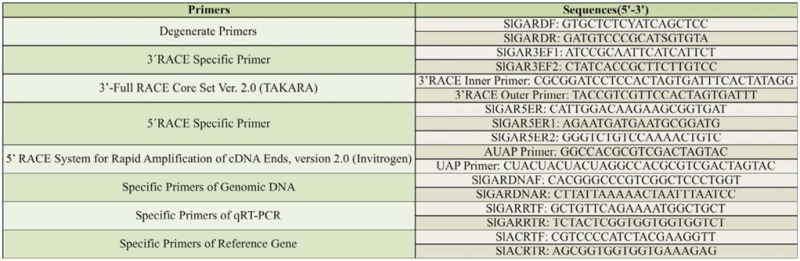
Primers used in the experiment.

## Materials and Methods

### Insect

*S. litura* of a laboratory susceptible strain were reared with artificial diet under conditions of 26 ± 1° C, 70–80% RH, and 16:8 L:D in a rearing room. The artificial diet contained 100 g of soybeans meal, 80 g of wheat bran, 8 g of casein, 8 g of vitamin C, 2 g of sorbic acid, 0.2 g of choline chloride, 0.2 g of inositol, and 0.2 g of cholesterol in 1 L volume.

### RNA preparation and synthesizing of firststrand cDNA

Fifth instar larvae of *S. litura* were ground in mortar with liquid nitrogen. Total RNA was extracted utilizing the Trizol Reagent (Omega, http://omegabiotek.com/) according to the protocol recommended by the manufacturer. 2 *µ* L total RNA was used as template to synthesize first-strand cDNA by PrimerScript Reverse Transcriptase and oligod(T)_18_ (TaKaRa, www.takara-bio.com).

### Partial sequence amplification of *SlGABARα2*

Degenerate primes SlGARDF and SlGARDR (Table 1) were designed according to the sequences of the GABA receptor in *B. mori* (NM001099824.1), *S. exigua* (EF535530.1), *P. xylostella* (FJ665610.1), and *Heliothis virescens* (AF006189.1) as reported on NCBI (www.ncbi.nlm.nih.gov). A partial sequence of *SlGABARα2* was amplified from firststrand cDNA of 5th instar larvae by SlGARDF and SlGARDR. Thermal cycling was done using the following protocol for touchdown PCR: 94° C for 3 min; 10 cycles at 94° C for 30 sec, annealing temperature decrease from 55 to 50° C for 30 sec, 2 cycles at each annealing temperature, 72° C for 1 min; another 20 cycles at 94° C for 30 sec, 50° C for 30 sec, 72° C for 1 min, and an additional polymerization step at 72° for 10 min. Amplified fragments were purified by 1.5% agarose gels, ligated into pMD 20-T vector (TaKaRa), transformed into *Escherichia coli* of DH5α to clone, and then positive clones were sequenced (Invitrogen, www.invitrogen.com).

### Rapid amplification of cDNA ends (RACE) of *SlGABARα2*

Specific primers SlGAR3EF1, SlGAR3EF2, SlGAR5ER, SlGAR5ER1, and SlGAR5ER2(Table 1) were designed according to the partial sequence of *SlGABARα2*, cloned as below to amplify the cDNA ends of *SlGABARα2* using Nested PCR. For 3′ end amplification, according to the instructions of 3′-Full RACE Core Set Version 2.0 (TaKaRa), the first round PCR used primers SlGAR3EF1 and Outer Primer with the following protocol: 94° C for 3 min; 30 cycles at 94° C for 30 sec, 55° C for 30 sec, 72° C for 2 min; and an additional polymerization step at 72° C for 10 min. The second round of PCR used primers of SlGAR3EF2 and Inner Primer, with the first round PCR product as the template. The thermal cycling protocol was as follows: 94° C for 3 min; 30 cycles at 94° C for 30 sec, 60° C for 30 sec, 72° C for 2 min; and an additional polymerization step at 72° C for 10 min. For 5′ end amplification, the 5′ RACE cDNA of *S. litura* was synthetized using primer SlGAR5ER according to the manufacturer's instructions of 5′ RACE System for Rapid Amplification of cDNA Ends, version 2.0 (Invitrogen). Subsequently, the first round PCR used primers SlGAR5ER1 and Abriged Anchor Primer with the following protocol: 94° C for 2 min; 30 cycles at 94° C for 30 sec, 55° C for 30 sec, 72° C for 1 min; and an additional polymerization step at 72° C for 10 min. The second round PCR used primers SlGAR5ER2 and UAUP Primer, with the first round PCR product as the template. The thermal cycling protocol was as follows: 94° C for 2 min; 30 cycles at 94° C for 30 sec, 60° C for 30 sec, 72° C for 1 min; and an additional polymerization step at 72° C for 7 min. The products of 3′ RACE and 5′ RACE were purified from 2.0% agarose gels and ligated into the T-vector (TaKaRa), then transformed into *Escherichia coli* of DH5α to clone, and positive clones were sequenced (Invitrogen).

### Genomic DNA isolation, and *SlGABARα2* DNA sequence amplification

Total genomic DNA was isolated from the adult *S. litura* according to the instruction of E.Z.N.A. Insect DNA Kit (OMEGA). Specific primers of genomic DNA SlGADNAF and SlGADNAR (Table 1) were designed according to the complete sequence of *SlGABARα2*. PCR reaction was done using TaKaRa LA Taq, which is particularly applicable to long sequence amplification. The PCR reaction was performed with the following protocol: 94° C for 3 min; 30 cycles at 94° C for 30 sec, 60° C for 30 sec, 72° C for 6 min; and an additional polymerization step at 72° C for 10 min. The amplified DNA that were isolated from the adult *S. litura* were purified by 1.5% agarose gels, then ligated into pMD 20-T vector (TaKaRa) and transformed into *E. coli* of DH5α to clone; then, positive clones were sequenced.

### Analysis of sequence structure and amino acid of *SlGABARα2*

Sequence similarity and analysis for conserved domains were performed using BLAST programs on NCBI. Amino acid sequences were derived and analyzed using EditSeq of DNASTAR 7.1 (www.dnastar.com). Signal peptide was analyzed by online software SignalP 3.0 Server(http://www.cbs.dtu.dk/services/SignalP/) (Dyrlov et al. 2004). Transmembrane domains prediction was performed with online software TMHMM Server v. 2.0 (http://www.cbs.dtu.dk/services/TMHMM/).

N-glycosylation, O-glycosylation, and phosphorylation sites was analyzed by NetNGlyc 1.0 Server, DictyOGlyc 1.1 Server, and NetPhos 2.0 Server (Center for Biological Sequence Analysis, www.cbs.dtu.dk) respectively (Julenius et al. 2005). The secondary structure of SlGABARα2 was analyzed using the online software PHD secondary structure prediction method (http://npsapbil.ibcp.fr/cgi-bin/secpred_phd.pl). Multiple sequence alignments were performed by Multalin version 5.4.1(http://multalin.toulouse.inra.fr/multalin/multalin.html). A phylogenetic tree was constructed using MEGA 5.05 (www.megasoftware.net) with the neighbor joining method and reconstructed with 1000 replicate bootstrap analysis.

### Homology modeling of SlGABARα2

The comparative modeling of the tertiary structure of SlGABARα2 was performed using the module of Build Homology Models of Discovery Studio 2.0 (Accelrys,www.accelrys.com). The template model that was applied for comparative modeling was searched in the 3D structure of Protein Data Bank (RCSB, www.rcsb.org/pdb). Ten models were predicted, and the model that attained the highest score was chosen as the final template model of SlGABARα2. The amino acid rationality of the constructed model was analyzed by Profile-3D and Ramachandran plots.

### Quantitative real-time PCR

The total RNA was exracted from eggs, 1st, 2nd, 3rd, 4th, 5th, and 6th instar larva, prepupae, 1st, 7th, and 14th day pupae, and adults. The brain, malpighian tube, midgut, epicuticle, and fat body of *S. litura* 6th instar larvae were extracted to examine relative transcription levels of *SlGABARα2* mRNA in different developmental stages and tissues using qRTPCR. The reaction was performed with BIORAD CFX96 Real-Time PCR Detection System (Bio-Rad, www.bio-rad.com) following the manufacturer's recommendations. The final volume of the 25 *µ*L reaction system contained 2 *µ*L cDNA (less then 100ng), 12.5 *µ*L SYBR *Premix Ex Taq*Tm (TaKaRa), 8.5 *µ*L ddH2O, 1 *µ* L of forward primer (10 *µ*M), and 1 *µ* L of reverse primer (10 *µ*M). qRT-PCR primers for *SlGABARα2* (SlGARRTF and SlGARRTR, Table 1) and *S. litura* β-actin protein (SlACRTF and SlACRTR, Table 1) were designed according to the complete sequence of *SlGABARα2* and *β-actin* cloned from *S. litura* respectively, and synthesized by the technical qRT-PCR primer design and synthesis company TaKaRa. The optimized real-time PCR protocol consisted of an initial step at 95° C for 30 sec followed by 40 cycles at 95° C for 5 sec, 60° C for 30 sec, and 15 sec at 72° C for extension and plate reading. After the cycling protocol, melting curves were obtained by increasing the temperature from 70° C to 95° C (0.4° C/sec) to denature the double-stranded DNA. All samples of real-time PCR were replicated 3 times, the dates were recorded, and data were analyzed by Bio-Rad CFX Manager. Quantification of the transcript level of *SlGABARα2* was conducted according to the 2^-ΔΔCt^ method (Livak and Schmittgen 2001), and statistical difference was determined followed by Duncan's Multiple Ranges Test method.

## Results

### Cloning and analysis of *SlGABARα2* complete sequence

A 370 bp fragment was amplified using degenerate primers SlGARDF and SlGARDR from the cDNA of 5th instar *S. litura*. The 5′ end (378 bp) and 3′ end (1399 bp) of *SlGABARα2* was amplified using RACE technique and the specific primers SlGAR3RF1, SlGAR3RF2, SlGAR5RR1, and SlGAR5RR2, respectively, which was designed based on the partial sequence of *SlGABARα2*. The complete sequence of *SlGABARα2* was spliced according to the partial 5′ end and 3′ end sequence by EditSeq of DNASTAR 7.1 and searched on the NCBI(www.ncbi.nlm.nih.gov) at amino acid level. The search result demonstrated that it shared 99.2% identity with GABA receptor a2 subunit gene from *S. exigua (SeGABARα2*, EF535530.1). It was named as *SlGABARα2*, and the GenBank accession number is JN792582.

**Figure 1.**
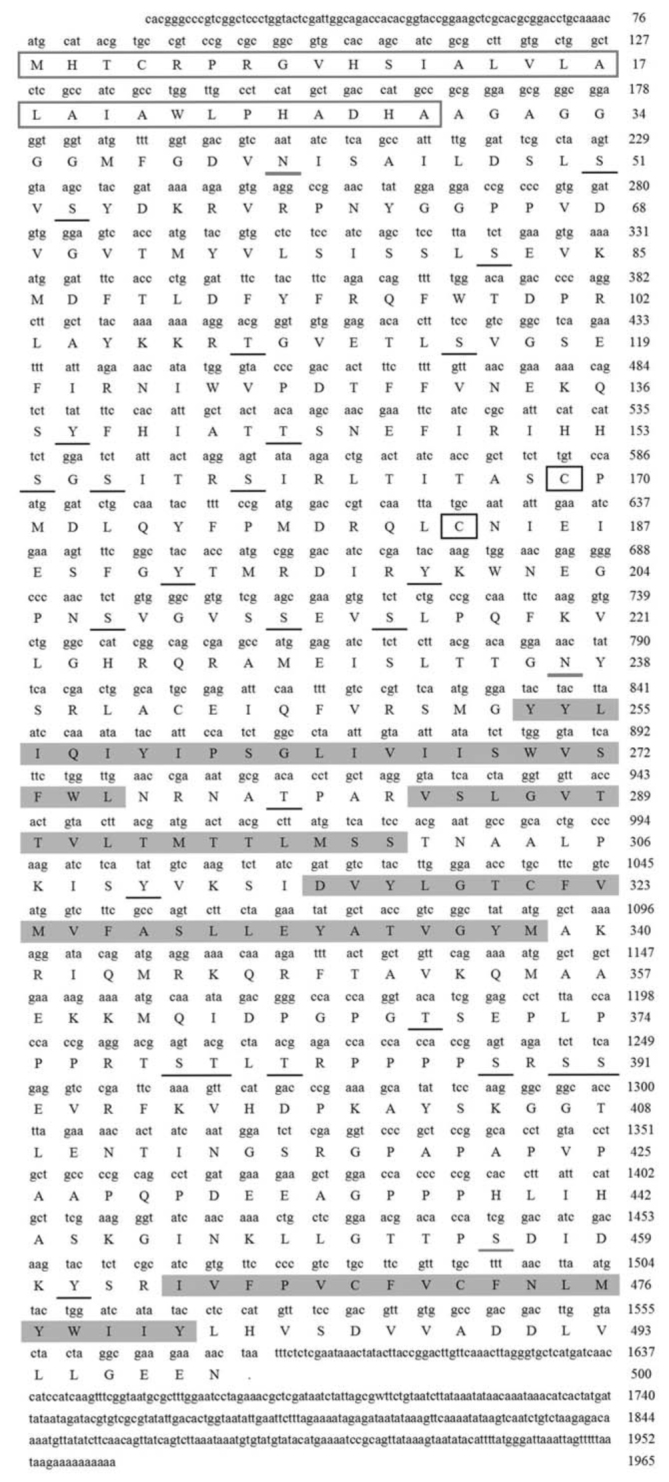
cDNA nucleotide sequence and deduced amino acid sequence of *Spodoptera litura SIGABARαL* Predicted N-terminal signal peptides were double framed; 2 N-glycosylation sites were treble underlined; 1 O-glycosylation site was double underlined; 30 phosphorylation sites were single underlined; I dicysteineloop was single framed; 4 transmembrane domains were shaded. High quality figures are available online.

**Figure 2.**
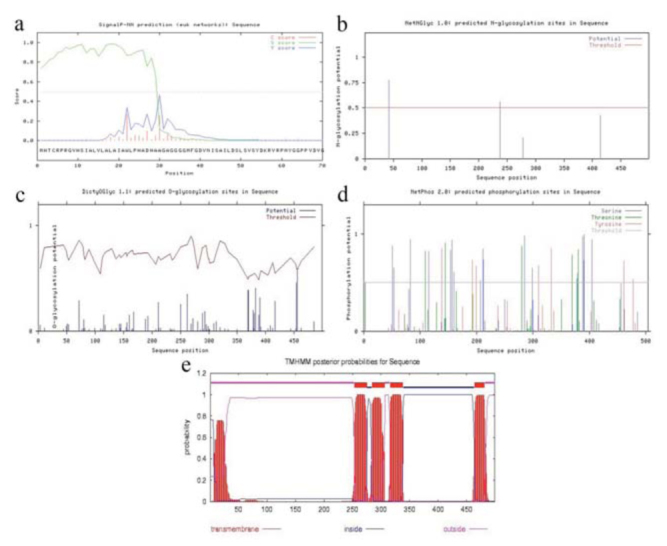
Analysis of the amino acid sequence of *Spodoptera litura* SlGABARα2. (a) Signal P prediction, (b) N-glycosylation site prediction, (c) O-glycosylation site prediction, (d) Phosphorylation site prediction. (e) Transmembrane domains prediction. High quality figures are available online.

**Figure 3.**
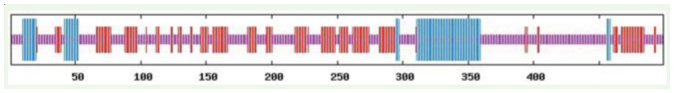
Secondary structure prediction of the amino acid sequence of *Spodoptera litura* SIGABARα2. Random coils are shown in purple, alpha helixes are shown in blue, extended strands are shown in red. High quality figures are available online.

The analysis of sequence structure and amino acids indicated that the full length of *SlGABARα2* had 1966 bp that consisted of a 76 bp 5′ end untranslated region, a 389 bp 3′ end untranslated region, and a 1500 bp open reading frame (Figure 1). The SlGABARα2 consisted of 499 amino acids, which had a computed molecular mass of 55.53 kD and predicted isoelectric point of 8.84. The amino acid sequence had a predicted N-terminal signal peptide consisting of 29 amino acids (Figure 2a), 2 N-glycosylation sites (Figure 2b), 1 O-glycosylation site (Figure 2c), 30 phosphorylation sites (Figure 2d), a highly conserved dicysteine-loop (Cys-loop), and 4 transmembrane domains (TM1, 253Y-275L; TM2, 284V-306S; TM3, 316D-338M; and TM4, 464I-48IY in SlGABARα2) (Figure 2e). Prediction of the SlGABARα2 secondary structure demonstrated that the amino acid sequence contained 15.43% α-helix, 30.46% extended strand, and 54.11% random coil (Figure 3).

### Homology modeling and rationality analysis of SlGABARα2

**Figure 4.**
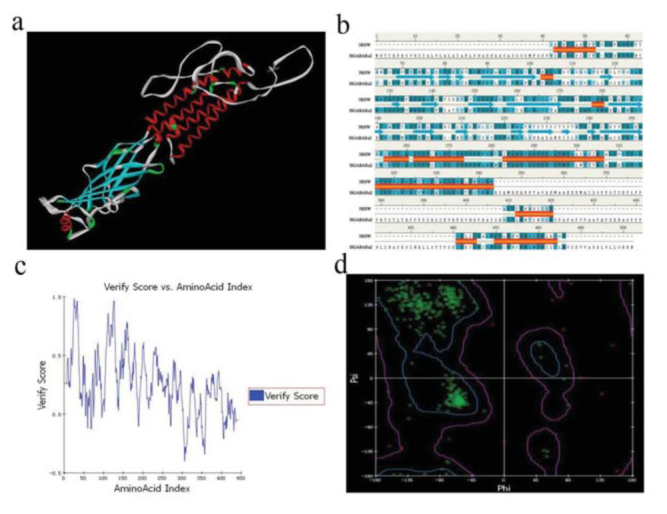
Prediction of the tertiary structure of *Spodoptera litura* SIGABARα2. (a) Comparative modeling of the 3D structure of SlGABARα2. α-helixes are shown in red, β-sheets are shown in blue, β-turns are shown in green, and irregular curls are shown in gray. (b) Comparative second structure in the tertiary model of SIGABAα2. α-helixes are shown in red bars, βsheets are shown in blue arrows, and irregular curls are shown in gray bars. (c) Profile-3D analysis on the 3D model of SlGABARα2. (d) Ramachandran plot analysis of SlGABARα2. Credible amino acids are marked with green plots and incredible amino acids are marked with red. High quality figures are available online.

The amino acid sequence of SlGABARα2 was blasted in the protein database. The search results indicated that the amino acid sequence of SlGABARα2 shared the highest homology (42%) with glutamate-gated chloride channel in *Caenorhabditis elegans* (protein database ID: 3RHW) (Hibbs and Gouaux 2011). The 3D structure of SlGABARα2 was constructed using the homology tertiary model of 3RHW (Figure 4a). The entire tertiary structure of SlGABARα2 was like a shuttle. It was mainly composed of 4 α-helixes (252G to 270W, 279T to 303A, 313S to 340K, and 458I to 781Y) (Figure 4b) on one side and some βsheets (67V to 249R) on the other side. The βturns and irregular curls were distributed in the entire model randomly. Interestingly, the 4 α-helixes were located at the 4 putative transmembrane domains, and β-sheets were located after the N-terminal signal peptide. Analysis of Profile-3D showed that the Verify Score of the amino acid index was mostly above zero except for amino acids at the C terminal (Figure 4c), which indicated that most amino acids of SlGABARα2 were located at rational position in the tertiary structure. Figure 4d shows 13 amino acids (red plots) in the disallowed region and 486 (97.4%) amino acids (green plots) in the allowed region. The results of Profile-3D and Ramachandran plots demonstrated that the tertiary structure of SlGABARα2 that was simulated by Build Homology Models of Discovery Studio 2.0 was logical.

### Analysis of multiple sequence alignments and the phylogenetic tree

**Figure 5.**
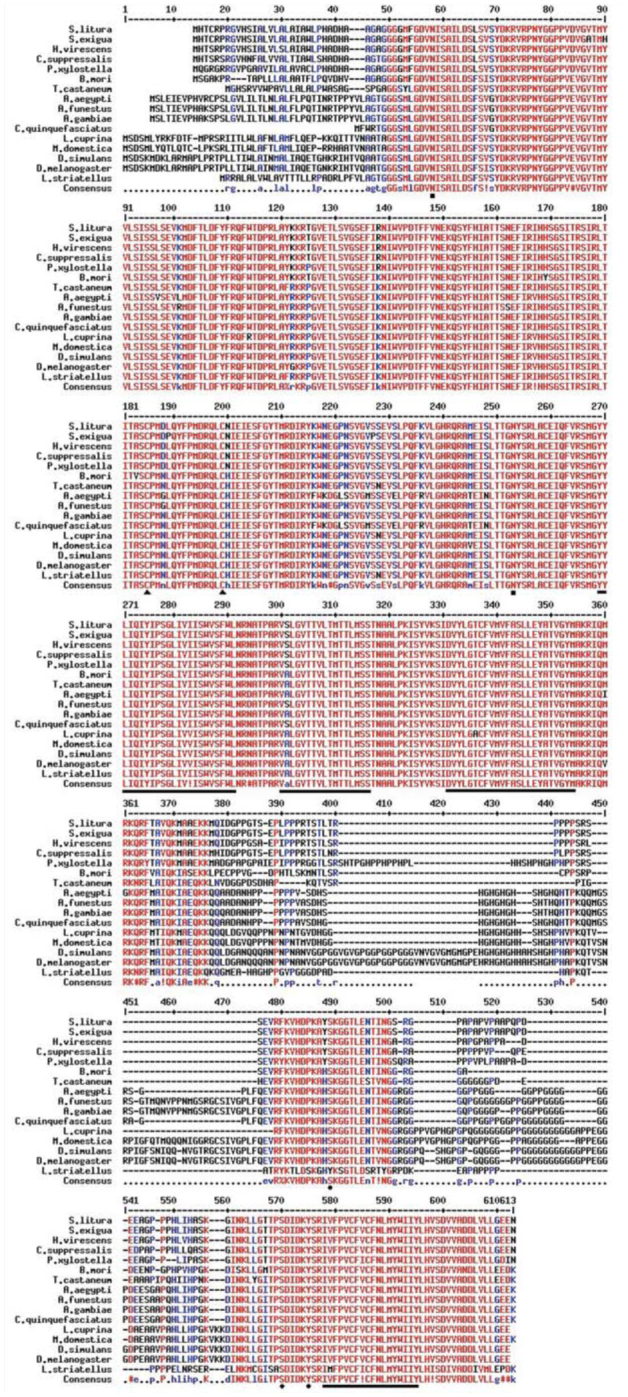
Multiple alignment of the amino acid sequence deduced from *Spodoptera litura SlGABARα2* with other GABA receptors in insects. Highly conserved N-phosphrylation, Oglycosylation site, and phosphrylation sites are marked by black squares, diamonds, and rotundity, respectively; The Cys-loop was marked by a black triangle; transmembrane domains were marked by black bars. The sequences were obtained from the GeneBank database, and the GeneBank accession numbers are as follow: *Spodoptera exigua:* EF535530.1, *Heliothis virescens:* AF006189.1, *Plutella xylostella:* FJ665610.1, *Bombyx mon:* NM001099824.1, *Chilo suppressalis:* HM566200.1, *Aedes aegypti:* U28803.1, *Anopheles funestus:* JF460792.1, *Anopheles gambiae:* XMOOI 688723.1, *Culex quinquefasciatus:* XM001850045.1, *Lucilia cuprina:* AF024647.1, *Musca domestica:* AB 177547.2, *Drosophila simulons:* AY017266.1, *Drosophila melanogaster*. U02042.1, *Laodelphax striatellus:* AB253526.1, *Tribolium castaneum:* NM001114337.1. High quality figures are available online.

**Figure 6.**
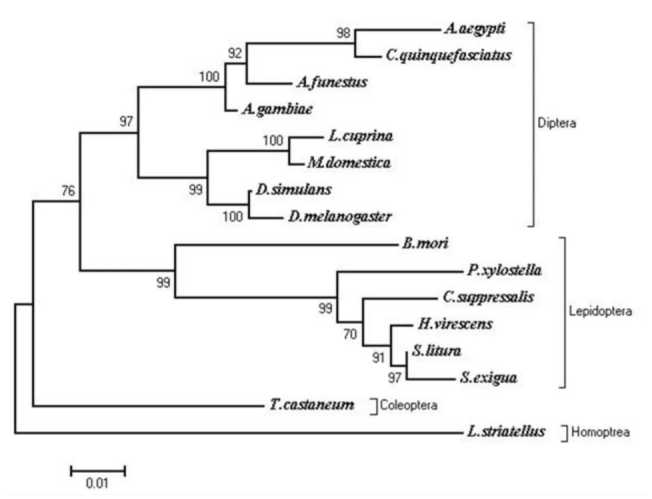
Phylogenetic tree of the GABA receptor in some insects. High quality figures are available online.

Multiple alignments of known amino acid sequences of GABA receptors were analyzed, and the results indicated that the sequences were highly conserved among different species of insects (Figure 5). The amino acid sequence of SlGABARα2 has the highest conversation degree among Lepidoptera, with 99.2% to *S. exigua*, 97.6% to *H. virescen*, 95.4% to *Chilo suppressalis*, 92.8% to *P. xylostella*, and 87.2% to *B. mori*. It also shares remarkable homology among Diptera, with 86.0% to *Culex quinquefasciatus*, 84.4% to *A. gambiae*, 84.0% to *Anopheles funestus*, 83.65% to *Musca domestica*, 82.0% to *Lucilia cuprina*, 82.0% to *Drosophila simulansv*, 81.7% to *Aedes aegypti*, and 81.6% to *D. melanogaster*. It has a relatively low identity to Coleoptera and Homoptera, with 83.6% to *T. castaneum* and 78.2% to *L. striatellus*, respectively. After comparing homological amino acid sequences of GABA receptors among different insects, it can be concluded that the highly conserved positions were located at 2 N-glycosylation sites (N42 and N237), 1 Oglycosylation site (456S), 2 phosphrylation sites (404S and 461Y), 1 dicysteine-loop (Cys-loop), and 4 transmembrane domains (TM1, TM2, TM3, and TM4) in SlGABARα2. The phylogenetic tree showed that the sequences of amino acid of GABA receptors had the nearest genetic distance in the same order (Figure 6). Lepidoptera, Diptera, Coleoptera, and Homoptera were well segregated from each other. The amino acid sequence of SlGABARα2 had the nearest genetic distance to the GABA receptor of *S. exigua* and the farthest genetic distance to the GABA receptor of *L. striatellus*. The genetic distances of the insects between Lepidoptera and Diptera were generally close.

### Genomic character of *SlGABARα2*

**Figure 7.**
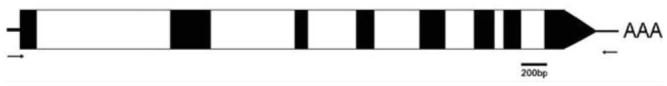
Schematic model of the genomic DNA of *Spodoptera litura SIGABARα2*. The lines at the 2 sides of the model indicate the 5′ and 3′ UTRs; black and white regions indicate exons and introns repectively; small arrows locating the positions of PCR primers for the amplifying of genomic sequence of *SIGABARα2*. High quality figures are available online.

The genomic DNA of *SlGABARα2* was amplified using the specific primers SlGARDNAF and SlGARDNAR from the total DNA of *S. litura* adults. The sequencing results demonstrated that the genomic DNA of *SlGABARα2* had a site of 5164 bp (Genbank accession number: JN794059) with 7 introns in it (Figure 7). These introns were located at 170 1531, 1924 - 2542, 2627 - 3093, 3232 - 3484, 3770 - 4010, 4193 - 4196, and 4439 - 4620 sites on the genomic DNA sequence of *SlGABAα2* respectively. All of these 7 introns were in accordance with the GT-AG splicing formula.

### Development- and tissue-specific expression patterns of *SlGABARα2*

**Figure 8.**
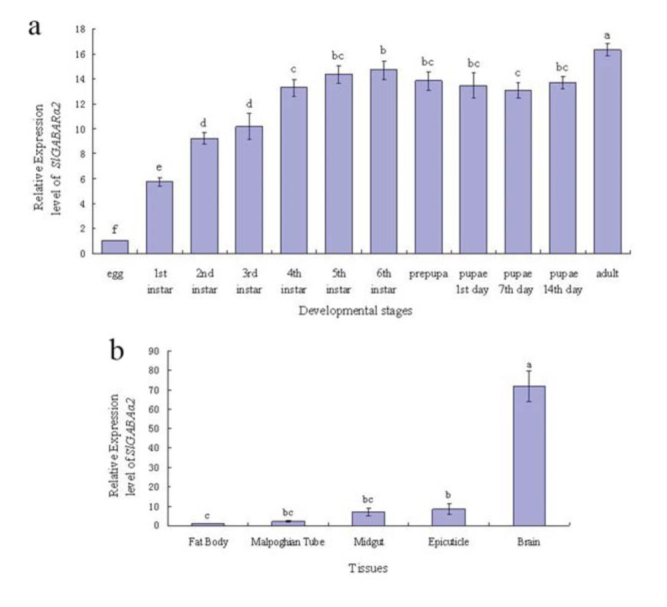
The relative expression levels of *Spodoptera SIGABARα2*. (a) The relative expression levels of *SIGABARα2* in different developmental stages of *S. litura*. (b) The relative expression levels of *SIGABARα2* in different tissue of *S. litura* larva. Each bar represents the mean ± SD of 3independent assays. The same letters above each bar indicate the expression level no significant difference in the different stages by DMRT (*p* = 0.05). High quality figures are available online.

The development- and tissue-specific expression patterns of *SlGABARα2* were detected using qRT-PCR. The results demonstrated that *SlGABARα2* was expressed in every development stage of *S. litura* (Figure 8a). The relative expression level of *SlGABARα2* in eggs was the lowest among the various development stages and increased with the larval growth. The relative expression level in 1st, 2nd, 3rd, 4th, 5th, and 6th instar larvae was 5.74, 9.25, 10.17, 13.29, 14.37, and 14.70fold higher than in eggs, respectively. However, the relative expression level in prepupae and 1st, 7th, and 14th day pupae was 13.84, 13.48, 13.10, and 13.69- fold higher than in eggs, respectively, which were a bit lower than in 6th instar. The highest relative expression level was in adults, which was 16.35-fold higher than in eggs. The relative expression level was remarkably dissimilar in the different tissues of *S. litura* larvae (Figure 8b). The lowest relative expression level tissue was the fat body. The relative expression level in the Malpighian tube, midgut, epicuticle, and brain was 2.57, 8.88, 10.93, and 79.34-fold higher than in the fat body, respectively.

## Discussion

The GABA receptor has been an important target for insecticides. It became the new focus of insect toxicology research because of the emergence of abamectin and fipronil, which target the GABA receptor in insects (Whiting 2003; Li et al. 2006). However, resistance to these insecticides appeared in many kinds of insects; for example, the sensibility to insecticides was reduced 100-fold in a resistant *D. melanogaster* population, the resistance being caused by a point mutation (Ala 302 to Ser) within the subunit gene *Rdl* of the GABA receptor (Ffrench-Constant et al. 1993; Anthony et al. 1998). A similar mutation was found for the resistant mechanism to fipronil in *P. xylostella* (Zhou et al. 2006). Therefore, further study on the molecular structure characters of GABA receptors is important to prevent resistance to these insecticides and develop novel, efficient, and safe insecticides that target the GABA receptor.

Studies of the GABA receptor subunits are still very limited, except for the RDL subunit. In the present study, the complete sequence of the GABA receptor α2 subunit in *S. litura* was cloned using the technology of RT-PCR and RACE. The amino acid of SlGABAα2 shares a high degree of conservation with those in other insects, especially those in Lepidoptera, which has the same N-terminal signal peptide at the initial position of the amino acid sequence. The different degrees of identity of GABA receptors among insects may be related to the genetic diversity. Multiple alignments of amino acid sequences of GABA receptors in different insects demonstrated that the highly conserved region was mainly present in positions after the N-terminal signal peptide of the amino acid sequence, such as the N-glycosylation, O-glycosylation, phosphorylation, and 4 transmembrane domains. Combining the result of multiple alignments of amino acid sequences and the simulated 3D structure of SlGABARα2, it is likely that the α2 subunit of GABA receptors in insects shares a similar tertiary structure that is embedded into the membrane by 4 transmembrane domains (α-helixes in the tertiary structure of SlGABARα2) that bind ions in other conserved domains (β-sheets in the tertiary structure of SlGABARα2) to transport them through the membrane. The DNA sequence information of *SlGABARα2* may helpto understand the feedback and regulation mechanism in some physiological process of *SlGABARα2* in *S. litura*.

The expression of *SlGABAα2* showed a distinct developmental- and tissue-specific pattern in *S. litura*. The expression level of *SlGABAα2* was the lowest in the eggs, which was probably due to the incomplete development of the nervous system in the embryo. It increased rapidly with larval growth, which is correlated with the rapid growth and complete development of tissues and the nervous system in the 4th, 5th, and 6th instar larvae. Perhaps the low expression level of *SlGABARα2* may be the reason why 1st, 2nd, and 3rd instar larvae are more sensitive to abamectin than 4th, 5th, and 6th instar larvae of *S. litura*. The relative expression level was reduced slightly in the pupal stage compared with 5th and 6th instar larvae and adults, which may be a result of the metamorphic development in the pupae when the nervous system was in the transformative process and the transportation of signals from the external was retarded. The tissue-specific pattern of *SlGABARα2* indicated that the GABA receptor was mainly expressed in the brain of the larvae, and was extremely low in the Malpighian tube, midgut, epicuticle, and fat body compared to the brain. These results are in accordance with the ditribution of the nervous system being mainly concentrated in the brain, while just a few neurocytes are distributed in other tissues, especially in the fat body. The characters of expression of the GABA receptor in *S. litura* suggest that the best period for controlling *S. litura* using pesticides targeting the GABA receptor is in the early instars. These results may be beneficial to develop some novel, efficient, and safe insecticides targeting the GABA receptor and to create new methods to restrain the GABA receptor in pests, such as RNA interference and pestresistant transgenic plants.

## Abbreviations:

GABA,: gamma-aminobutyric;
qRT-PCR,: quantitative real time PCR;
RACE,: rapid amplification of cDNA ends;
RT-PCR: reverse transcription PCR;
*SICABARα*2.: the mRNA of *Spodoptera litura* gamma-aminobutyric receptor α2 subunit;
SIGABARα2,: the protein of *Spodoptera litura* gamma-aminobutyric receptor α2 subunit;
**TM**,: transmembrane
